# ﻿The six whole mitochondrial genomes for the *Diaporthe* species: features, evolution and phylogeny

**DOI:** 10.3897/imafungus.16.140572

**Published:** 2025-02-28

**Authors:** Shunpei Xie, Xuyang Ma, Haiyan Wu, Rui Zang, Haiqiang Li, Miao Liu, Qiang Li, Qingzhou Ma, Yashuang Guo, Meng Zhang

**Affiliations:** 1 Department of Plant Pathology, Henan Agricultural University, Zhengzhou, Henan, China; 2 Institute of Plant Protection, Xinjiang Academy of Agricultural Sciences, Urumqi, China; 3 College of Agronomy, Henan Agricultural University, Zhengzhou, Henan, China; 4 School of Food and Biological Engineering, Chengdu University, Chengdu, Sichuan, China

**Keywords:** Comparative analysis, *Diaporthe*, gene rearrangement, intron, Mitogenome, phylogenetic analysis

## Abstract

In this study, the complete mitogenomes of three *Diaporthe* species (*Diaportheeres* ZM79-3, *D.phaseolorum* ZM33-4 and *Diaporthe* sp. ZM41-5) were sequenced, assembled and compared with the other three previously sequenced *Diaporthe* mitogenomes (*D.caulivora* VNIIKR SE Dcaul3, *D.longicolla* MSPL 10-6 and *D.sojae* VNIIKR SE Dps12). The six *Diaporthe* mitogenomes were found to be circular DNA molecules, with lengths ranging from 53,646 bp to 108,865 bp. The mitogenomes of the six *Diaporthe* species mainly comprised the same set of 15 core protein-coding genes (PCGs), two rRNAs, and a certain number of tRNAs and unidentified open reading frames (ORFs). The PCG length, AT skew and GC skew showed large variability among the 15 PCGs in the six mitogenomes. The *nad1* gene had the least K2P genetic distance of the 15 core PCGs among the 13 *Diaporthales* species, indicating that this gene was highly conserved. The Ka/Ks values for all 15 core PCGs were < 1, suggesting that these genes were all subject to purifying selection. Comparative mitogenome analysis showed that introns contributed the most to the size variation of *Diaporthe* mitogenomes. Frequent intron loss/gain events were detected to have occurred in the *cox1* gene during the evolution of the *Diaporthales* mitogenomes. Although the mitogenomes of 13 species from *Diaporthales* had undergone large-scale gene rearrangements, six mitogenomes of *Diaporthe* species had identical gene arrangements. Phylogenetic analysis based on combined mitochondrial gene datasets showed that the six *Diaporthe* species formed well-supported topologies. To our knowledge, this study is the first report on the mitogenomes of *D.phaseolorum* ZM33-4 and *Diaporthe* sp. ZM41-5, as well as the first comparison of mitogenomes among *Diaporthe* species. Our findings will further promote investigations of the genetics, evolution and phylogeny of the *Diaporthe* species.

## ﻿Introduction

The genus *Diaporthe* was originally established with *D.eres* by Nitschke in 1870, which has been discovered worldwide on a wide variety of host plants, functioning as endophytes, pathogens, and saprobes (Nitschke 1870; Gomes et al. 2013), and was accommodated in the *Diaporthaceae* (*Diaporthales*, *Sordariomycetes*; Hyde et al. 2014). In the past, a number of crucial plant diseases caused by *Diaporthe* species were reported over time. *D.eres* has a wide range of plant hosts, including blueberry (Lombard et al. 2014), pear (Bai et al. 2015), grapes (Lawrence et al. 2015), and hazelnut (Arciuolo et al. 2021). *D.sojae*, *D.phaseolorum*, *D.longicolla* and *D.caulivora* were first reported and act as important pathogens on pods and stems of soybean (Santos et al. 2011; Udayanga et al. 2015). In addition, *D.sojae* was also reported on some fruit trees in China, such as *Vitis* spp. (Dissanayake et al. 2015), *Pyrus* spp. (Guo et al. 2020) and *Citrus* spp. (Xiao et al. 2023).

Currently, *Diaporthe* is a large and taxonomically complex genus with over 1200 species recorded in Index Fungorum (https://www.indexfungorum.org/names/Names.asp). Many members of *Diaporthe* were traditionally considered difficult to classify because of the lack of reliable morphological characteristics, the overabundance of synonyms, and the widespread misuse of names (van Rensburg et al. 2006; Udayanga et al. 2011; Dissanayake et al. 2020). Consequently, the introduction of molecular markers has greatly promoted our taxonomic classification of *Diaporthe* (Santos et al. 2017; Guo et al. 2020). Multi-gene phylogenetic analyzed utilizing gene markers such as the internal transcribed spacer region (ITS), translation elongation factor 1-alpha (*tef*), beta-tubulin (*tub*), calmodulin (*cal*), and histone H3 (*his*) have proven to be effective tools for both the identification of *Diaporthe* species and the study of evolutionary relationships among them (Udayanga et al. 2012). However, many novel species have been reported based on this method over the past years, leading to an expansion in the number of species within *Diaporthe* (Norphanphoun et al. 2022; Bhunjun 2023). Dissanayake et al. (2024) recently divided the genus *Diaporthe* into seven sections based on the five-locus dataset (ITS, *tef*, *tub*, *cal*, and *his*) and supported by GCPSR (Genealogical Concordance Phylogenetic Species Recognition) methodology and coalescence-based models, and proposed boundaries for 13 species and 15 species-complexes (Dissanayake et al. 2024). This changed the taxonomic framework of *Diaporthe* greatly, which shows that simple five-gene phylogenetic analysis without safeguarding via aforementioned principles is insufficient for species-level resolution. In recent decades, the mitochondrial genome (mitogenome) has emerged as a reliable and effective molecular marker for phylogenetic analyses of eukaryotes (Li et al. 2019b; Abuduaini et al. 2021; Song et al. 2024). With the rapid advancement of next-generation sequencing technology, increasing numbers of mitochondrial genome sequences have been acquired (https://www.ncbi.nlm.nih.gov/genome/browse#!/organelles/), which has greatly enhanced our understanding of the phylogenetic relationships among eukaryotes, and provided a more comprehensive source of genetic information (Li et al. 2018c; Li et al. 2019a; Anwar et al. 2023).

Mitochondria are double membrane organelles, well-known for their major role in energy supply, which are believed to be acquired from alpha-proteobacteria by eukaryotic ancestors through endosymbiosis (Muñoz-Gómez et al. 2017). Because of its many advantages, including maternal inheritance, rapid evolution, low recombination rates, and many available molecular markers, the mitochondrial genome has become a useful tool for the study of taxonomy, phylogeny, evolution, population genetics, and comparative genomics (Burger et al. 2003; Bullerwell and Lang 2005; Wang et al. 2020a). However, compared with animals and plants, the fungal mitogenome has been less studied (Richards et al. 1998), especially that of *Sordariomycetes*. To date, less than 90 complete mitogenomes of *Sordariomycetes* can be accessed in public databases, compared to the innumerable *Sordariomycetes* in nature (*Sordariomycetes* mitogenome – Nucleotide – NCBI (nih.gov)). The available mitochondrial genomes of *Sordariomycetes* are very limited and were reported to greatly vary in mitochondrial gene content, structure, genome size and gene order in previous reports (Yuan et al. 2019; Aguileta et al. 2014). In addition, introns, homing endonuclease genes, plasmid-derived genes, repetitive sequences, tRNAs, and genes transferred from the nuclear genome are thought to have led to dynamic changes in the structure and size of the fungal mitogenomes (Férandon et al. 2010; Himmelstrand et al. 2014; Kanzi et al. 2016; Wang et al. 2020b), which also provided abundant information for understanding the origin and evolution of fungi. Despite large variations in mitochondrial content, most fungal species were shown to contain 14 conserved protein coding genes (*atp6*, *atp8*, *atp9*, *cob*, *cox1*, *cox2*, *cox3*, *nad1*, *nad2*, *nad3*, *nad4*, *nad4L*, *nad5*, and *nad6*) for energy metabolism, and one conserved ribosomal protein S3 gene (*rps3*) for transcriptional regulation (Osiewacz 2002; Chatre and Ricchetti 2014; Yuan et al. 2017). Besides, there are small and large ribosomal RNAs and a set of tRNA genes in the fungal mitogenome (Li et al. 2018b). To date, only four genomes derived of *Diaporthe* species that can be considered mitochondrial-complete were obtainable in the NCBI database, of which two mitogenomes (*D.longicolla* MSPL 10-6 and *D.nobilis* NIE8444; note: *D.nobilis* as synonymous to the *D.eres*) were reported in publications and the other two mitogenomes (*D.caulivora* VNIIKR SE Dcaul3 and *D.sojae* VNIIKR SE Dps12) remained so-far unpublished (Koloniuk et al. 2016; Eo 2021; Li et al. 2024). Although these results contribute to our initial understanding of the mitogenome characteristics of the genus *Diaporthe*, the number of the *Diaporthe* mitogenomes is so small which greatly limits our understanding of its genetic evolution. Additionally, up until now, no interspecific comparative analysis has been attempted for *Diaporthe*.

In the present study, the mitogenomes of three *Diaporthe* phytopathogens (*D.eres* ZM79-3, *D.phaseolorum* ZM33-4 and *Diaporthe* sp. ZM41-5) were sequenced, assembled, annotated, and compared with the other three available *Diaporthe* species mitogenomes (*D.caulivora* VNIIKR SE Dcaul3, *D.longicolla* MSPL 10-6 and *D.sojae* VNIIKR SE Dps12) from the NCBI database. The aims of the study were: 1) to characterize the mitogenome contents, structures and organizations of the *D.phaseolorum* ZM33-4 and *Diaporthe* sp. ZM41-5; 2) to perform comparative mitogenomic analysis of the *Diaporthales* species and reveal their variations and conservations; 3) to investigate the intron dynamic changes of *cox1* genes in 13 *Diaporthales* species including the six *Diaporthe* species; 4) to clarify the phylogenetic status of *Diaporthe* in the *Ascomycota* phylum based on the combined mitochondrial gene set. This comparative analysis of *Diaporthe* mitogenomes will contribute to a deeper understanding of genetic evolution and species differentiation within the *Diaporthe* genus.

## ﻿Material and methods

### ﻿Fungal isolates, DNA extraction and genome sequencing

Utilizing the method of tissue isolation (Rizali et al. 2021), *D.eres* ZM79-3, *D.phaseolorum* ZM33-4 and *Diaporthe* sp. ZM41-5 strains were obtained from branches and trunks of apple trees in Zhengzhou, Henan province, China. The hyphal tips were transferred onto PDA plates for purification, and after approximately 3 days of incubation at 25 °C, the culture plates were stored at 4 °C for short-term preservation. For long-term storage, the fungal colonies were incubated for about 7 days, and edges of the colonies were removed into frozen tubes, which were then stored in 25% glycerol at -80 °C in the fungal collection of Henan Agricultural University. Total genomic DNA (gDNA) extraction of the three isolates was performed by using the cetyltrimethyl ammonium bromide (CTAB) method (Chen et al. 2020). *Diaporthe* species were identified based on morphological characters and five-locus dataset of ITS, *TUB*, *CAL*, *TEF1-α*, *HIS* genes (Guo et al. 2020). These sequences have been deposited in GenBank with the accession numbers listed in Suppl. material 1: table S1. The associated taxonomic study will be published in a separate study.

High-quality gDNA samples were then sent to the Novogene Co., Ltd. (Tianjin, China) for library preparation and genome sequencing. Short insert libraries (350 bp) were created using the NEBNext® Ultra DNA Library Prep Kit for Illumina (NEB, USA). Whole-genome sequencing (WGS) was performed on an Illumina Hiseq X Ten platform, producing 150 bp paired-end reads for each sample.

### ﻿Mitogenomes assembly and annotation of *Diaporthe* species

Approximately 5 Gb of raw data were obtained through WGS, and were then processed by trimming adapters and low-quality reads (bases with a Q20 ratio > 30% or containing undetermined bases) using the fastp v0.13.1 (Chen et al. 2018). Subsequently, duplicate removal and error correction were carried out using FastUniq v1.1 (Xu et al. 2012) and Musket v1.1 (Liu et al. 2013) sequentially. The cleaned paired-end reads were assembled *de novo* using the SPAdes v3.14.1 software with kmers of 21, 33, 55, 77, 99, and 127 (Bankevich et al. 2012). Mitochondria-related contigs were then identified and pooled for each sample by performing BLASTN search against the reference mitogenomes from *D.longicolla* MSPL 10-6 (Koloniuk et al. 2016). The gaps between these contigs were filled using MITObim v1.9 to construct closed-circular mitochondrial DNA (mtDNA) macromolecules of the three *Diaporthe* samples (Hahn et al. 2013). Furthermore, the three assembled mitochondrial sequences were verified using NOVO Plasty (Dierckxsens et al. 2017).

The newly obtained complete three mitogenomes (*D.eres* ZM79-3, *D.phaseolorum* ZM33-4 and *Diaporthe* sp. ZM41-5), along with three *Diaporthe* mitogenomes (*D.caulivora* VNIIKR SE Dcaul3, *D.longicolla* MSPL 10-6 and *D.sojae* VNIIKR SE Dps12) obtained from the NCBI GenBank database, were meticulously annotated following the methods described by Ma et al. (Ma et al. 2022). Initially, Mfannot (Valach et al. 2014) and MITOS (Bernt et al. 2013) were used to predict the protein coding genes (PCGs), unidentified open reading frames (uORFs), rRNAs, tRNAs, and introns of the six *Diaporthe* mitogenomes based on genetic code 4 (mold mitochondrial). Subsequently, the NCBI Open Reading Frame Finder (https://www.ncbi.nlm.nih.gov/orffinder) was employed to modify or predict the PCGs and uORFs, which were further annotated through BLASTP searches against the NCBI non-redundant protein sequence database (Bleasby and Wootton 1990). The intron and exon boundaries of PCGs were verified using the exonerate v2.2 software (Slater and Birney 2005) with *D.longicolla* MSPL 10-6 mitogenomes as references (Koloniuk et al. 2016). Secondary structures of the tRNA genes were predicted by MITOS with default parameters (Bernt et al. 2013), and were further verified by utilizing tRNAscan-SE v2.00 (https://lowelab.ucsc.edu/tRNAscan-SE/index.html), and were redrawn in Adobe Illustrator CS6. The graphical maps of the six *Diaporthe* mitogenomes were created using the online program OGDraw v1.2 (Greiner et al. 2019).

For further comparative analyses of *Diaporthales* mitogenomes, other seven complete mitogenomes of *Diaporthales* species were downloaded from the NCBI GenBank database (Kanzi et al. 2016). Their detailed information (such as accession numbers) was provided in Suppl. material 1: table S2.

### ﻿Sequences analyses and repetitive elements

The strand asymmetries of the thirteen *Diaporthales* mitogenomes were assessed based on the formulas: AT skew = [A − T] / [A+T], and GC skew = [G − C] / [G + C] (Zou et al. 2022). Mitogenome collinearity analysis of the six *Diaporthe* species was performed using Mauve v2.4.0 (Darling et al. 2004). The sequence manipulation suite was utilized to analyze the codon usage frequency and preference of the six *Diaporthe* mitogenomes based on genetic code 4 (Stothard 2000). The KaKs Calculator 3.0 software (Zhang 2022) was used to calculate synonymous (*Ks*) and nonsynonymous substitution rates (*Ka*) for 15 core PCGs (*atp6*, *atp8*, *atp9*, *cob*, *cox1*, *cox2*, *cox3*, *nad1*, *nad2*, *nad3*, *nad4*, *nad4L*, *nad5*, *nad6* and *rps3*) in all 13 acquired *Diaporthales* mitogenomes. Based on the Kimura-2-parameter (K2P) substitution model, MEGA v6.06 was used to calculate the genetic distances between each pair of the 15 core PCGs (Caspermeyer 2016). Additionally, BLASTN searches were conducted within the six *Diaporthe* mitogenomes to detect interspersed repeats or intragenomic duplications of large fragments with an E-value of <1e-10 (Chen et al. 2015). Tandem Repeats Finder was used to analyze the tandem repeats of six *Diaporthe* mitogenomes (Benson 1999). In addition, the contribution rates of different genetic compositions to the mitogenome expansion/contraction of six *Diaporthe* species were calculated using the formulas: [A − B] / [C − D] (A: size of one genetic component in the larger mitogenome; B: size of the same genetic component in the smaller mitogenome; C: size of the larger mitogenome; D: size of the smaller mitogenome).

### ﻿Comparative mitogenomic and intron analyses of Diaporthales species

A comparative mitogenomic analysis was performed to evaluate the variations and conservations between *Diaporthales* species in terms of genome size, base composition, GC content, gene number, intron number, gene arrangement, and gene content. As mobile genetic elements in mitogenome, introns could significantly alter the organization and size of fungal mitogenomes, which can be divided into different position classes (Pcls) based on their precise insertion position in the coding region of core genes (Ma et al. 2022). Following the method described by Férandon et al. (Férandon et al. 2010), introns within the *cox1* genes of the 13 *Diaporthales* mitogenomes could be categorized into different position classes (Pcls). Using the *cox1* gene of *Juglanconisjuglandina* mitogenome as the reference (Kanzi et al. 2016), the *cox1* genes from the other 12 *Diaporthales* species were aligned with Clustal W to detect the insertion sites of introns (Thompson et al. 1994). Pcls were named according to their insertion sites in the corresponding reference sequence. Generally, the same Pcls from different species exhibited high sequence similarity and contained homologous intronic ORFs (Megarioti and Kouvelis 2020).

### ﻿Mitochondrial phylogenomic analysis

In order to investigate the phylogenetic relationships of the six *Diaporthe* species, a phylogenetic tree was constructed with 98 *Ascomycetes* species using the concatenated mitochondrial gene set, which included 14 core PCGs and the *rps3* gene. *Taphrinadeforman* and *T.wiesneri* from *Taphrinomycetes* were appointed as outgroups (Tsai et al. 2014). Mitochondrial genes were aligned using Clustal W in MEGA v6.06 software (Caspermeyer 2016), and the FASconCAT v1.0 tool was used to combine 15 genes into a concatenated mitochondrial gene set (Kück and Meusemann 2010). Partition homogeneity test was used to detect potential phylogenetic conflicts between different mitochondrial genes, and PartitionFinder v2.1.1 was applied for determination of best-fit models of phylogeny and partitioning scheme of the gene set (Lanfear et al. 2017). Subsequently, we utilized Bayesian inference (BI) and maximum likelihood (ML) methods for phylogenetic analysis. BI analysis was conducted with MrBayes v.3.1.2 using the GTR substitution model and gamma-distributed rate variation for the site-to-site variance ratio, and four Markov chain Monte Carlo (MCMC) chains were analyzed twice using 2 × 10^7^ generation random trees. During the analysis, samples were taken every 1000 generations and stopped when the mean standard deviation of the splitting frequency fell below 0.01, discarding the 25% aged samples from the obtained samples, and the remaining trees were used to calculate Bayesian posterior probability (BPP) values in the 50% majority-rule consensus trees (Ronquist and Huelsenbeck 2003; Shapiro et al. 2006). The ML analysis was performed in IQtree v.1.6.8 under the GTR + G substitution model. Bootstrap values (BS) were assessed with 1000 replicates using an ultrafast bootstrap approach (Hillis and Bull 1993; Nguyen et al. 2015). Phylogenetic trees were visualized and adjusted with FigTree v1.4.4 (http://tree.bio.ed.ac.uk/software/figtree/).

### ﻿Data availability

The complete three mitogenomes of *D.eres* ZM79-3, *D.phaseolorum* ZM33-4 and *Diaporthe* sp. ZM41-5 were deposited in the GenBank database under the accession numbers PQ493439, PQ493438 and PQ493440, respectively, and their raw sequencing data were deposited in the Sequence Read Archive (SRA) database under the accession numbers SRR31801374, SRR31801985 and SRR31806477, respectively.

### ﻿Abbreviations

**Mitogenome**: Mitochondrial genome; **PCG**: Protein-coding gene; **Pcls**: Position classes; ***Ks***: Synonymous substitution rates; ***Ka***: Nonsynonymous substitution rates; **BI**: Bayesian inference; **ML**: Maximum Likelihood; **ITS**: internal transcribed spacer; ***tef1***: translation elongation factor-1 alpha; ***tub***: beta-tubulin; ***his***: histone; ***cal***: calmodulin.

## ﻿Results

### ﻿Features, compositions and PCGs of *Diaporthe* mitogenomes

**Figure 1.**
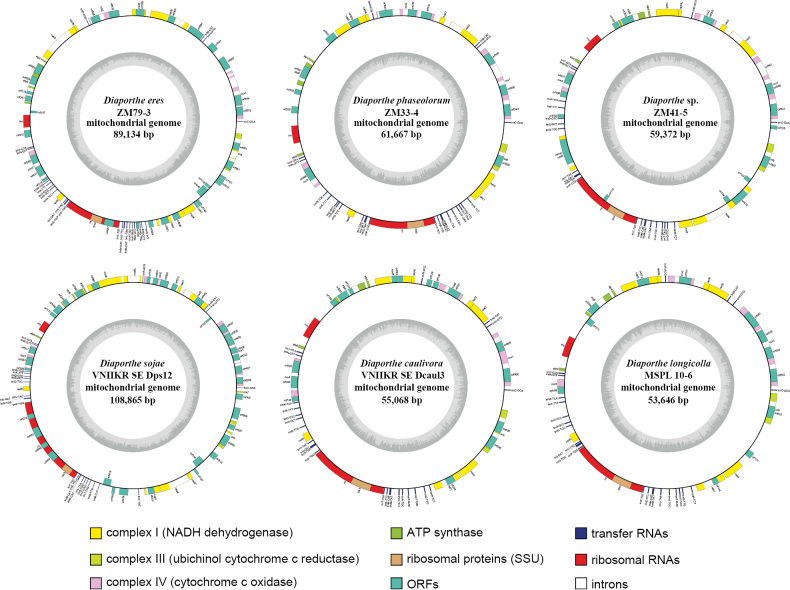
Circular maps of the six *Diaporthe* mitogenomes. Genes are represented by different colored blocks. Colored blocks outside each ring indicate that the genes are on the forward strand, while colored blocks within the ring indicates that the genes are located on the reverse strand. Genes on the forward strand are transcribed in a counterclockwise direction, while genes on the reverse strand are transcribed in a clockwise direction. The inner grayscale bar graph shows the GC content of the mitochondrial sequences. The circle inside the GC content graph marks the 50% threshold.

In the present study, the complete mitochondrial genomes of the three *Diaporthe* species (*D.eres* ZM79-3, *D.phaseolorum* ZM33-4 and *Diaporthe* sp. ZM41-5) were circularly assembled with the total sizes of 89,134 bp, 61,667 bp and 59,327 bp, respectively. In addition, three *Diaporthe* mitogenomes (*D.caulivora* VNIIKR SE Dcaul3, *D.longicolla* MSPL 10-6 and *D.sojae* VNIIKR SE Dps12) were downloaded from public databases with sizes of 55,068 bp, 53,646 bp, and 108,865 bp, respectively, and compared and analyzed with the three newly assembled *Diaporthe* mitogenomes (Fig. 1). The GC content of the six *Diaporthe* mitogenomes ranged from 32.30% to 34.63%, with an average GC content of 33.24% (Suppl. material 1: table S2). The mitogenome of *Diaporthe* sp. ZM41-5 contained the highest GC content, while *D.eres* ZM79-3 contained the lowest GC content, among the six investigated species. The six *Diaporthe* mitogenomes all contained both positive GC skew and negative AT skew. The proportion of protein-coding regions, intronic regions, intergenic regions, and ncRNA regions (including tRNAs and rRNAs genes) are shown in Suppl. material 2: fig. S1. In the mitochondrial genomes of *D.eres* ZM79-3, *D.sojae* VNIIKR SE Dps12, and *Diaporthe* sp. ZM41-5, intronic regions and protein-coding regions accounted for more than 59% of the whole mitogenomes and intergenic regions accounted for 26.09%–27.84% of the entire mitogenomes. In the other three *Diaporthe* mitogenomes, intergenic regions and protein-coding regions occupied the largest proportion, accounting for more than 60% and intronic regions accounting for 21.83–26.57% of the whole mitogenomes. Their ncRNA genes (including tRNAs and rRNAs) took up the smallest proportion of the six *Diaporthe* mitogenomes, reaching only 8.99%–15.03%.

The number of PCG identified in the mitogenomes of the six *Diaporthe* species ranged from 18 to 28 (Suppl. material 1: table S2). The *D.sojae* VNIIKR SE Dps12 mitogenome was found to contain the most PCG among all six mitogenomes detected. Moreover, all six *Diaporthe* mitochondrial genomes contained 14 typical core PCGs (*atp6*, *atp8*, *atp9*, *cob*, *cox1*, *cox2*, *cox3*, *nad1*, *nad2*, *nad3*, *nad4*, *nad4L*, *nad5*, and *nad6*) and a conserved core gene coding for the putative ribosomal protein S3 (*rps3*). In addition, some PCGs in the mitogenomes of the six *Diaporthe* species were found to encode homing endonucleases (HEs). Some of the PCGs in the six detected mitogenomes were not similar to any known proteins in public databases, which indicates that there are still a variety of PCGs in the *Diaporthe* mitochondrial genomes that have yet to be characterized. A number of introns were also detected ranging from 10 to 29 in the six mitogenomes, with *D.sojae* VNIIKR SE Dps12 containing the highest number of introns and *D.caulivora* VNIIKR SE Dcaul3 and *D.longicolla* MSPL 10-6 the lowest (Suppl. material 1: tables S2, S3). Meanwhile, 23 intronic ORFs were found in the mitogenome of *D.sojae* VNIIKR SE Dps12, 11 in *D.caulivora* VNIIKR SE Dcaul3, 17 in *D.eres* ZM79-3, 8 in *D.longicolla* MSPL 10-6, 10 in *D.phaseolorum* ZM33-4 and 11 in *Diaporthe* sp. ZM41-5. These intronic ORFs mainly encoded HEs with LAGLIDADG and GIY-YIG endonuclease motifs, and a few encoded proteins with unknown function (Suppl. material 1: table S3).

### ﻿rRNA genes, tRNA genes, and codon analyses of the six *Diaporthe* mitogenomes

The mitogenomes of six *Diaporthe* species both contained two rRNA genes, namely the small subunit ribosomal RNA (*rns*), and the large subunit ribosomal RNA (*rnl*) (Suppl. material 1: table S3). The lengths of *rnl* genes in the six detected mitogenomes ranged from 5,952 bp to 14,526 bp and 1,611 bp to 1,727 bp in the *rns* genes. The longest *rnl* was found in the mitogenome of *D.sojae* VNIIKR SE Dps12 and the longest *rns* was found in the mitogenome of *D.caulivora* VNIIKR SE Dcaul3 (Suppl. material 1: table S3). The average lengths of the *rnl* and *rns* genes were 7,744 bp and 1,647 bp, respectively. In addition, we also found that the *rnl* genes had 1–4 introns in the six *Diaporthe* species, while no introns were detected in the *rns* genes.

**Figure 2.**
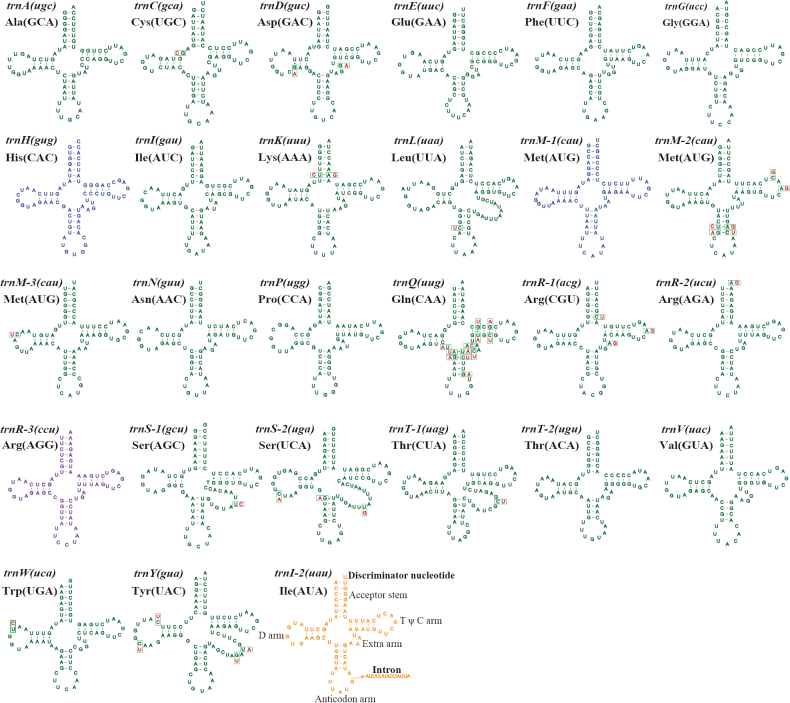
Putative secondary structures of tRNA genes identified in the mitogenomes of six *Diaporthe* species. The 23 tRNAs in green fonts represent tRNAs shared by the six *Diaporthe* species, while the tRNAs in blue font represent in all *Diaporthe* species except *Diaporthe* sp. ZM41-5, the tRNA in orange font represents tRNA only in *D.sojae* VNIIKR SE Dps12, and the tRNA in purple font represents tRNA only in *D.eres* ZM79-3. Besides, residues conserved across the six mitogenomes are shown in green, while variable sites are shown in red.

We detected 23 to 26 tRNA genes in the six *Diaporthe* mitogenomes, which encoded 20 standard amino acids (Suppl. material 1: table S3). All six mitogenomes contained three tRNAs that coded for arginine, serine and threonine with different anticodons and two tRNAs that coded for methionine with the same anticodons. We also detected additional tRNA genes, including the *trnH*, *trnM*, *trnR*, and *trnY* in the six mitogenomes (Fig. 2). The size of the tRNA genes ranged from 71 bp to 86 bp, mainly due to variation in the size of the extra arms (Suppl. material 1: table S3). In addition, we also observed an interesting phenomenon in which a 13-base intron sequence was inserted between the sequences of the antisense codon arm of the *trnI-2* in the *D.sojae* VNIIKR SE Dps12 mitogenome, resulting in a length of 83 bp for this gene. Of the 23 tRNA genes shared by the six *Diaporthe* mitogenomes, 14 contained mutational sites. A total of 41 mutational sites were detected in the all tRNA genes among the six *Diaporthe* mitogenomes (Fig. 2 and Suppl. material 1: table S4).

**Figure 3.**
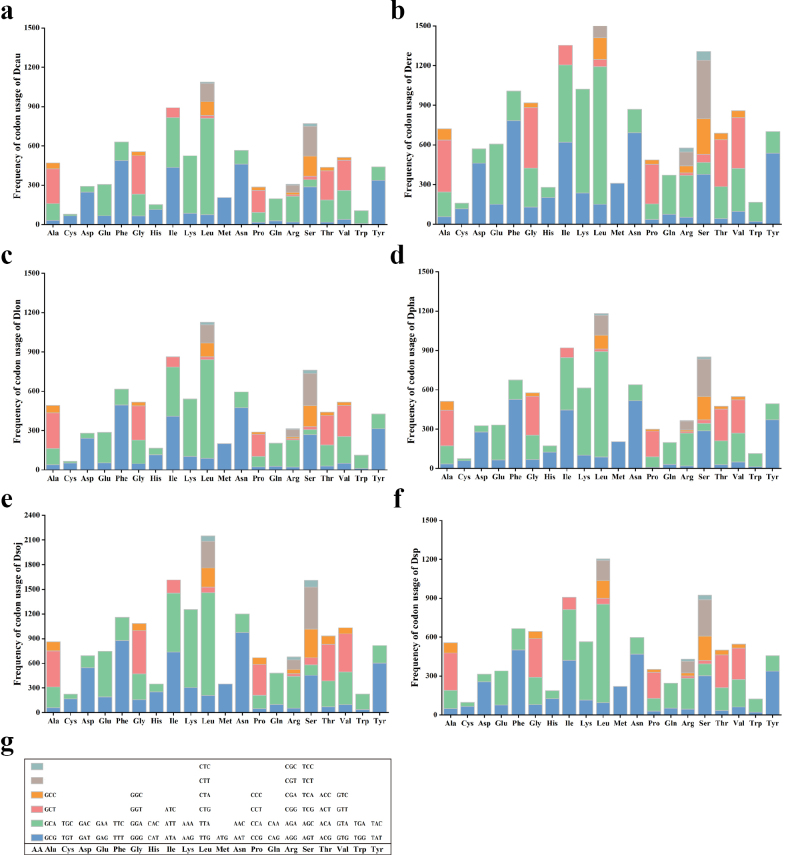
Codon usage in the mitogenomes of six *Diaporthe* species. Frequency of codon usage is plotted on the y-axis **a***Diaporthecaulivora* VNIIKR SE Dcaul3 **b***D.eres* ZM79-3 **c***D.longicolla* MSPL 10-6 **d***D.phaseolorum* ZM33-4 **e***D.sojae* VNIIKR SE Dps12 **f***Diaporthe* sp. ZM41-5 **g** genetic code 4 (mold mitochondrial).

Most core PCGs in the six *Diaporthe* mitogenomes used ATG as start codons, with an exception of the *cox3* gene, which used TGT as start codons in all the six *Diaporthe* species (Suppl. material 1: table S5). TAA (72.38%) was the most common used stop codon in the core PCGs of six *Diaporthe* mitogenomes, followed by TAG (27.62%). The stop codons varied among the six *Diaporthe* species. i.e., the *atp6* genes of *D.eres* ZM79-3, *D.sojae* VNIIKR SE Dps12, *Diaporthe* sp. ZM41-5, *D.caulivora* VNIIKR SE Dcaul3 and *D.phaseolorum* ZM33-4 used TAA as a stop codon, while *D.longicolla* MSPL 10-6 used TAG as a stop codon. The nad6 gene of *D.phaseolorum* ZM33-4 had TAA as a stop codon, and the *nad6* genes of the other six species used TAG as a stop codon (Suppl. material 1: table S5). Codon usage analysis showed that the most frequently used codons in the six *Diaporthe* mitogenomes were TTA (for Leucine; Leu), TTT (for phenylalanine; Phe), AAA (for lysine; Lys), AAT (for asparagine; Asn), and ATA (for isoleucine Ile) (Fig. 3 and Suppl. material 1: table S6). The high frequency of use of A and T in codons resulted in the high AT content of the six *Diaporthe* mitogenomes (average 66.76%).

### ﻿Overlapping nucleotides and intergenic regions

We have identified overlapping nucleotides in all six *Diaporthe* mitochondrial genomes, and the highest degree of overlap was found in the *D.caulivora* VNIIKR SE Dcaul3 mitogenome, which had three overlapping nucleotides (Suppl. material 1: table S3). Overlapping nucleotides were all observed in the six *Diaporthe* mitogenomes, located across the adjacent genes *nad4L* and *nad5* (−1 bp). The length of each overlapping nucleotide in the six mitogenomes ranged from 1 bp to 202 bp. The largest overlapping nucleotide among the six *Diaporthe* mitogenomes was detected in *D.longicolla* MSPL 10-6 and *Diaporthe* sp. ZM41-5, which was located between *cox3* and *orf118* (−202 bp) and between *cox3* and *orf168* (−202 bp), respectively. Intergenic region sequences ranging from 16,527 bp to 28,755 bp were detected in the six *Diaporthe* mitogenomes, with occupations ranging from 26.09% to 33.05%. The lengths of these intergenic segments ranged from 1 to 2,590 bp, with the longest intergenic region located between *orf170* and *cob* in the mitogenome of *D.longicolla* MSPL 10-6 (Suppl. material 1: table S3).

### ﻿Repeat elements analysis

Using BLASTN searches of the six *Diaporthe* mitogenomes against themselves, we identified 39, 44, 47, 67, 45, and 20 repetitive elements in the mitogenomes of *D.caulivora* VNIIKR SE Dcaul3, *D.eres* ZM79-3, *D.longicolla* MSPL 10-6, *D.phaseolorum* ZM33-4, *D.sojae* VNIIKR SE Dps12 and *Diaporthe* sp. ZM41-5, respectively (Suppl. material 1: table S7). The size of these repeat elements ranged from 36 to 1,283 bp, with pairwise nucleotide similarities ranging from 64.15% to 100%. The largest repeat region was found in the mitogenome of *D.sojae* VNIIKR SE Dps12, which was located in the protein coding regions of *orf474* and intergenic sequences around it, as well as in the protein coding regions of *orf518* and intergenic sequences around it. Repetitive sequences accounted for 4.38%, 10.99%, 6.54%, 8.24%, 19.48%and 2.74% of the *D.caulivora* VNIIKR SE Dcaul3, *D.eres* ZM79-3, *D.longicolla* MSPL 10-6, *D.phaseolorum* ZM33-4, *D.sojae* VNIIKR SE Dps12 and *Diaporthe* sp. ZM41-5 mitogenomes, respectively (Suppl. material 1: table S7).

Tandem repeat sequences accounted for 0.67%, 0.98%, 0.32%, 1.74%, 2.00% and 1.41% of the *D.caulivora* VNIIKR SE Dcaul3, *D.eres* ZM79-3, *D.longicolla* MSPL 10-6, *D.phaseolorum* ZM33-4, *D.sojae* VNIIKR SE Dps12 and *Diaporthe* sp. ZM41-5 mitogenomes, respectively. 8, 15, 4, 10, 26, and 13 tandem repeats were identified in *D.caulivora* VNIIKR SE Dcaul3, *D.eres* ZM79-3, *D.longicolla* MSPL 10-6, *D.nobilis*, *D.phaseolorum* ZM33-4, *D.sojae* VNIIKR SE Dps12 and *Diaporthe* sp. ZM41-5 mitogenomes, respectively (Suppl. material 1: table S8). In addition, the length of repeat units ranged from 3 bp to 168 bp, and the copy number ranged from 1.9 to 22.5, and the longest tandem repeat sequence (168 bp) was detected in the mitogenome of *D.sojae* VNIIKR SE Dps12, which was located in the protein coding regions of *orf458* (Suppl. material 1: tables S3, S8).

### ﻿Intron dynamics of *cox1 gene* in Diaporthales

A total of 315 introns were detected in the 13 *Diaporthales* mitogenomes, each of which contained 10 to 48 introns. Most introns in *Diaporthales* belonged to the Group I. The intron number varied greatly in the *Diaporthales* mitogenomes which indicated that intron acquire/loss events have occurred in the evolution of *Diaporthales* species. The *cox1* gene of *Diaporthales* contained the most number of introns, accounting for 31.11% (98/315) of the total introns. Thus, intron dynamics in the *cox1* gene was used for further analysis among all the 13 *Diaporthales* species.

**Figure 4.**
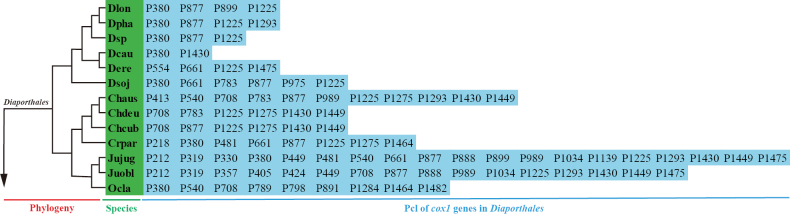
Position class (Pcl) information of *cox1* genes in the 13 *Diaporthales* species. Introns in *cox1* genes of 13 mitogenomes calibrated were classified into different position classes (Pcls) using the *cox1* gene of *Juglanconisjuglandina* as the reference. Each Pcl was constituted by introns inserted at the same position of corresponding *cox1* gene of *J.juglandina* and named according to its insertion site in the aligned corresponding reference sequence (nt). The Pcls in different color represent their different numbers among the 13 *Diaporthales* mitogenomes. Phylogenetic positions of the 13 species were established using the Bayesian inference (BI) method and Maximum-Likelihood (ML) methods based on combined mitochondrial data sets.

A total of 35 different Pcl types were detected in *cox1* genes of 13 *Diaporthales* species (Suppl. material 2: fig. S2). The number of introns varied greatly between species, with the highest number of Pcls (19) in the *cox1* gene of *J.juglandina* and the lowest number of Pcls (2) in *D.caulivora* VNIIKR SE Dcaul3 (Fig. 4). It should be emphasized that the *cox1* gene of *J.juglandina* contained the same number of introns as the maximum number of introns (19) reported for the *Agaricus bisporus cox1* gene, which has the largest number of introns ever found in the *cox1* gene (Férandon et al. 2010). Here, 35 Pcls were identified widely distributed in the 13 *Diaporthales* species. Among them, P380, P877 and P1225 were the most common Pcls, which were detected 8, 10 and 12 times in 13 *Diaporthales* species, respectively. Pcls, including P218, P330, P357, P405, P413, P424, P554, P789, P798, P975, P1139, P1284, P1438 and P1482 could only be detected in one of the 13 *Diaporthales* mitogenomes. These results indicated that ancestors of *Diaporthales* lost or gained introns to a large extent during evolution. Additionally, 23 Pcls were detected in the *cox1* genes of six *Diaporthe* species, accounting for 23.47% of the total introns in the *cox1* gene of 13 *Diaporthales* species. *D.sojae* VNIIKR SE Dps12 had the highest number of Pcls (6) and *D.caulivora* VNIIKR SE Dcaul3 had the lowest number of Pcls (2). Meanwhile, Pcls P380 and P1225 were found to be distributed in five of the six *Diaporthe* species, and P877 was detected in four of the six *Diaporthe* species, suggesting that these Pcls might be widespread in *Diaporthe* species.

### ﻿Genetic distance, evolutionary rates and variation of 15 core PCGs

**Figure 5.**
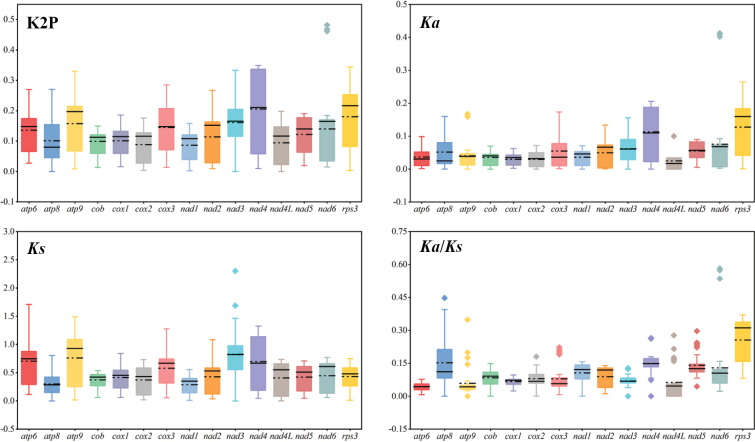
Genetic analysis of 15 core protein coding genes (including *rps3* gene) in 13 *Diaporthales* species. The black straight and dotted lines indicate the magnitude of the median and mean values, respectively. K2P, pairwise genetic distances between each pair of the 15 core PCGs in the 13 *Diaporthales* mitogenomes based on the Kimura-2-parameter model; *Ka*, the number of nonsynonymous substitutions per nonsynonymous site; *Ks*, the number of synonymous substitutions per synonymous site.

Here, we used 15 core PCGs to calculate the genetic distance and substitution rates between each pair of 13 *Diaporthales* species. The *nad4* gene had the greatest genetic distance (average value 0.24) among the 13 *Diaporthales* species, followed by the *rps3* (average value 0.20), which indicated that they showed the fastest mutation rate among the 15 PCGs. The *nad1* gene exhibited the lowest genetic differentiation between the 13 *Diaporthales* species, with an overall mean K2P distance of 0.10, indicating that the *nad1* gene was highly conserved (Fig. 5 and Suppl. material 1: table S5). Across the 15 core PCGs examined, the *rps3* gene exhibited the highest non-synonymous substitution rate (Ka) (average value 0.14) between the 13 *Diaporthales* mitogenomes, followed by *nad4* (average value 0.13), while the *nad4L* gene had the lowest (average value 0.03). The synonymous substitution (Ks) rate of *nad3* was the highest (average value 0.97), and that of *nad1* was the lowest (average value 0.32) among the 15 PCGs. The overall Ka/Ks values for 15 examined core PCGs were less than 1, which suggested that these genes were subject to purifying selection (Suppl. material 1: table S5).

**Figure 6.**
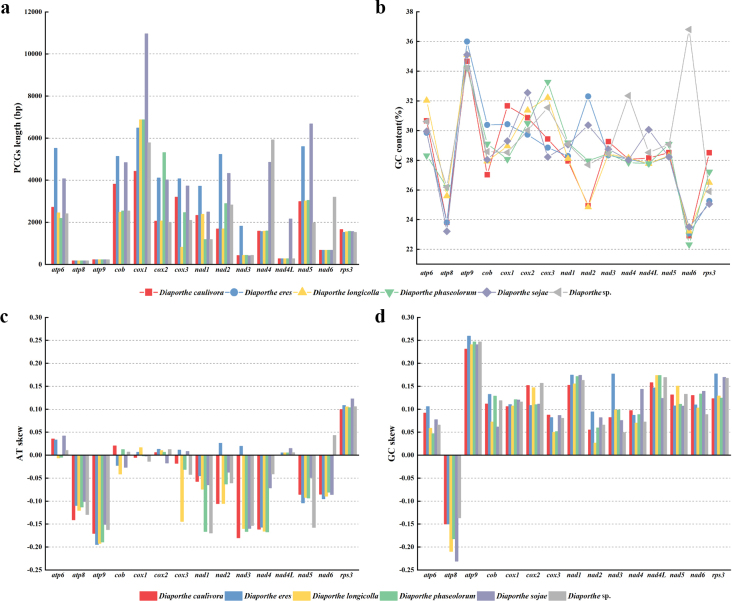
Full-length sequence variation in the length and base composition of each of the 15 protein coding genes (PCGs) among six *Diaporthe* mitochondrial genomes **a** PCG length variation **b** GC content across PCGs**c** AT skew **d** GC skew.

In addition, we further conducted an in-depth comparison of the similarities and differences between the 15 core PCGs among the six *Diaporthe* species. Among the 15 core PCGs, the length of the 13 PCGs varied significantly except for the *atp8* and *atp9* genes, which had identical gene lengths in the six *Diaporthe* species, with the *cox1* gene having maximum length variation of 5,183 bp (Fig. 6). Across the 15 core PCGs we detected, *atp9* contained the highest average GC content of 34.89%, followed by *cox2* with 30.83%. The *atp8* gene contained the lowest GC content with an average of 24.80%. Additionally, we found that the same PCGs also had differences in GC content among the six mitogenomes. i.e., *nad6* contained the highest GC content of 36.81% in *Diaporthe* sp. ZM41-5, while in other five *Diaporthe* species GC content in *nad6* gene only ranged from 22.32% to 23.51% (Suppl. material 1: table S5). These differences indicated that the core PCGs of *Diaporthe* mitogenomes mutated frequently. The AT skews of *atp8*, *atp9*, *nad1*, *nad4*, and *nad5* in the six mitogenomes were negative, while the AT skew of *rps3* was positive (Fig. 6). The AT skew of *nad4L* was zero in *D.caulivora* VNIIKR SE Dcaul3 and the AT skew of *cox2* was negative in *D.sojae* VNIIKR SE Dps12, while the AT skews of *nad4L* and *cox2* were positive in the other five *Diaporthe* species. The *nad6* showed positive AT skew in *Diaporthe* sp. ZM41-5 and negative AT skew in the other five *Diaporthe* species. The *nad2* and *nad3* showed positive AT skew in *D.eres* ZM79-3 and negative AT skew in the other five *Diaporthe* mitogenomes. The *atp6* exhibited negative AT skew in *D.longicolla* MSPL 10-6 and *D.phaseolorum* ZM33-4 and positive AT skew in the other four *Diaporthe* species. The *cob* exhibited positive AT skew in *D.caulivora* VNIIKR SE Dcaul3, *D.phaseolorum* ZM33-4 and *Diaporthe* sp. ZM41-5. and negative AT skew in the other three *Diaporthe* species. The *cox1* showed positive AT skew in *D.eres* ZM79-3 and *D.longicolla* MSPL 10-6, and negative AT skew in the other four *Diaporthe* species. The *cox3* exhibited positive AT skew in *D.eres* ZM79-3 and *D.sojae* VNIIKR SE Dps12 and negative AT skew in the other four *Diaporthe* species. AT skew in core PCGs varied among species within the genus, indicating frequent A/T mutations in core PCGs. The GC skews were positive in most core PCGs, with an exception of *atp8*, which had negative GC skew (Suppl. material 1: table S5).

### ﻿Mitochondrial gene arrangement in Diaporthales species

**Figure 7.**
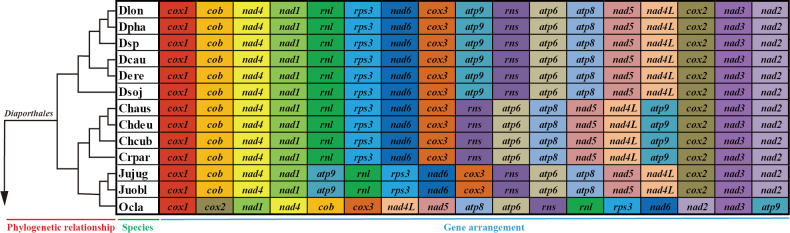
Mitochondrial gene arrangement analyses of the 13 *Diaporthales* species. The gene sequence begins with the *cox1* gene and contains 15 core protein coding genes (PCGs) and two rRNA genes. The same genes were represented by the same color blocks. Phylogenetic positions of the 13 species were established using the Bayesian inference (BI) method and Maximum-Likelihood (ML) method based on combined mitochondrial data sets.

In the present study, we analyzed the gene arrangements of 15 core PCGs and 2 rRNAs in the 13 *Diaporthales* mitogenomes, and found that there were large variations in gene arrangement among different genera, except for the genus *Cryphonectria* and *Chrysoporthe* which had identical gene arrangement (Fig. 7). Besides, three uninterrupted conserved gene blocks, *cob*, *nad4* and *nad1* (Block I), *rns*, *atp6*, *atp8*, *nad5* and *nad4L* (Block II), *rnl*, *rps3* and *nad6* (Block III), were also found in the mitochondrial gene arrangement of 13 *Diaporthales* species. Meanwhile, we also observed the identical gene arrangements between species from the same genus, such as *Diaporthe*, *Juglanconis* and *Chrysoporthe*.

Whole-mitogenome collinearity analysis was performed between six closely related *Diaporthe* species. A total of 4 locally collinear blocks (A to D) were detected in each of the six *Diaporthe* mitogenomes using Mauve (Suppl. material 2: fig. S3). The size of these homologous regions varied among different species, when compared to the *D.sojae* VNIIKR SE Dps12 mitogenome, most of these homologous region blocks in the other five species were significantly reduced in size. Except for the rearrangement of homologous regions B and C in the mitogenome of *D.eres* ZM79-3, the other homologous regions were in the same order among the six *Diaporthe* species. In summary, the gene arrangement and collinearity in the mitogenomes of the six *Diaporthe* species were almost highly conserved within the genus.

### ﻿Comparative mitogenomic analysis and phylogenetic analysis

Across the 13 *Diaporthales* species examined, there was a large variation in the mitogenome sizes, which ranged from 53,646 to 267,504 bp, with an average size of 125,149 bp (Suppl. material 1: table S2). *J.juglandina* had the largest mitogenome (267,504 bp), and *D.longicolla* MSPL 10-6 had the smallest (53,646 bp). The sizes of all the six *Diaporthe* mitogenomes (53,646 bp to 108,865 bp) were smaller than the average mitogenome size of the 13 *Diaporthales* species tested (125,419 bp). Meanwhile, the GC content of the 13 mitochondrial genomes also varied, ranging from 29.71 to 34.63%, with an average GC content of 32.46%. The average GC content of the six *Diaporthe* species (33.24%) was higher than the average GC content of the 13 *Diaporthales* mitogenomes, and the *Diaporthe* sp. ZM41-5 mitogenome contained the highest GC content among the 13 mitogenomes tested. All the 13 *Diaporthales* mitogenomes had positive GC skews, while only 2 of the 13 mitogenomes had positive AT skews (Suppl. material 1: table S2). The number of PCGs in the 13 *Diaporthales* mitogenomes ranged from 18 to 49, with *Chrysoportheaustroafricana* containing the highest number of PCGs and *D.longicolla* MSPL 10-6 and *D.phaseolorum* ZM33-4 containing the lowest. The *J.juglandina* mitogenome had the largest numbers of introns (48) and intronic ORFs (83) among the 13 *Diaporthales* species, followed by the *J.oblonga* mitogenome with 39 introns and 58 intronic ORFs. In addition, two rRNA genes and 23–28 tRNA genes were detected in the 13 *Diaporthales* mitogenomes (Suppl. material 1: table S2).

**Figure 8.**
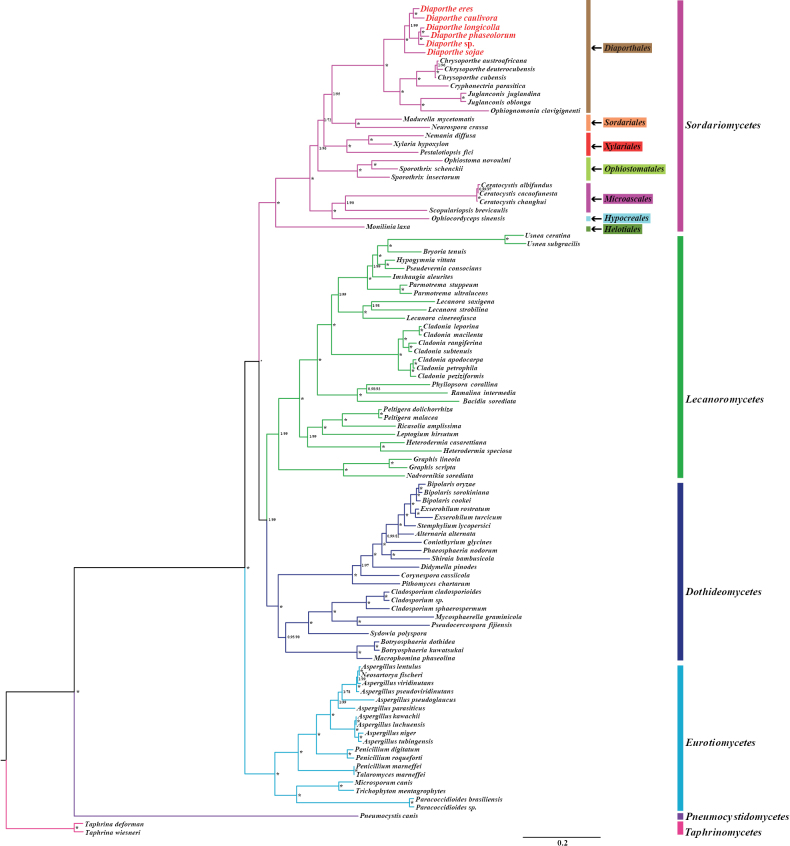
Molecular phylogeny of 98 *Ascomycota* species based on Bayesian inference (BI) and Maximum likelihood (ML) analysis of 15 core protein coding genes. Support values are Bayesian posterior probabilities (BPP) and bootstrap values (BS) placed before and after the slash, respectively. Asterisks indicate BPP and BS values of 1.00 and 100, respectively. Both *Taphrinadeforman* and *T.wiesneri* from *Taphrinomycetes* were appointed as the outgroup. Species and NCBI registry numbers of mitogenomes used for phylogenetic analyses can be provided in Suppl. material 1: table S9.

To clarify the phylogenetic status of *Diaporthe* in the phylum *Ascomycota*, an identical and well-supported phylogenetic tree for 98 *Ascomycota* species was constructed using both Bayesian inference (BI) and Maximum likelihood (ML) methods based on the 15 concatenated mitochondrial conserved PCG genes (Fig. 8). We found all the major clades had good supported values within the phylogenetic tree (BPP ≥ 0.95; BS ≥ 72). Both *T.deforman* and *T.wiesneri* from *Taphrinomycetes* were designated as the outgroup, while the other 96 *Ascomycota* species were divided into five major clades, corresponding to *Dothideomycetes*, *Sordariomycetes*, *Lecanoromycetes*, *Eurotiomycetes* and *Pneumocystidomycetes* (Suppl. material 1: table S9). Within the *Sordariomycetes*, members of *Diaporthales* were also well separated from members of neighboring *Microascales*, *Ophiostomatales*, *Xylariales*, *Sordariales*, and *Hypocreales*. Among the 13 *Diaporthales*, the different members were well separated with strong BS support and BPP values of 1 for all clades. Particularly, members of the five genera *Diaporthe*, *Juglanconis*, *Ophiognomonia*, *Cryphonectria* and *Chrysoporthe* were shown to closely cluster together in the inferred tree, indicating a close relationship. Additionally, members of the same genus clustered together, conforming their generic relationship as sister species in *Diaporthe*. Overall, phylogenetic analyses highlighted the potential of mitogenomes in aiding taxonomists to differentiate between members of *Diaporthe* and its closely related genera.

## ﻿Discussion

### ﻿Mitogenome size variation in *Diaporthe* species

In the present study, we newly sequenced and assembled the mitogenomes of *D.eres* ZM79-3, *D.phaseolorum* ZM33-4 and *Diaporthe* sp. ZM41-5 and compared them with three *Diaporthe* mitogenomes published previously, namely of *D.caulivora* VNIIKR SE Dcaul3, *D.longicolla* MSPL 10-6 and *D.sojae* VNIIKR SE Dps12, as well as seven other *Diaporthales* species (Kanzi et al. 2016; Koloniuk et al. 2016; Li et al. 2024). The size of the 13 examined *Diaporthales* mitogenomes varied largely, with the largest belonging to *J.juglandina*, which was 4.99 times greater than the smallest mitogenome (*D.longicolla* MSPL 10-6). Significant genomic size variability was also observed among the mitogenomes of the six *Diaporthe* species examined, particularly between *D.sojae* VNIIKR SE Dps12 and *D.longicolla* MSPL 10-6, with a range of 102.93% ((max-min)/min) (Suppl. material 1: table S2). Previous studies had indicated that fungal mitogenome expansion/contraction was closely linked to the accumulation of repetitive sequences, plasmid-derived genes, intergenic regions, and intron dynamics (Boussau et al. 2011; Zubaer et al. 2018; Chen et al. 2021). In this study, using the mitogenome of *D.longicolla* MSPL 10-6 as a reference, we found that intron regions contributed the most to mitochondrial expansion of *D.caulivora* VNIIKR SE Dcaul3, *Diaporthe* sp. ZM41-5, *D.phaseolorum* ZM33-4, *D.eres* ZM79-3 and *D.sojae* VNIIKR SE Dps12 with 101.41%, 89.33%, 58.33%, 58.56%, and 50.79%, respectively (Suppl. material 2: fig. S1). This result was consistent with previous research indicating the significant role of intron regions in the process of fungal mitogenome size changes (Li et al. 2019b; Li et al. 2020; Ma et al. 2022; Song et al. 2024). Similarly, using the mitogenome of *D.sojae* VNIIKR SE Dps12 as a reference, we also found that the intronic and intergenic regions played primary and secondary roles in the contraction processes in all *Diaporthe* species. Our results also highlighted the importance of intergenic regions in *Diaporthe* mitochondrial size changes. Repetitive sequences also contributed to the expansion/contraction process, although their contribution rates were lower than those of intronic regions. No plasmid-derived genes were identified in any of the six *Diaporthe* mitogenomes, similar to findings in the mitogenomes of the *Cryphonectria* and *Chrysoporthe* genus (Kanzi et al. 2016). In summary, the sequencing of the three new *Diaporthe* mitogenomes in this study, along with the three previously sequenced *Diaporthe* mitogenomes, enhanced our understanding of mitogenome size boundaries and variations within the *Diaporthe* genus (Koloniuk et al. 2016; Li et al. 2024).

### ﻿Gene content variation in *Diaporthe* species

During long-term evolution, the mitogenome of fungi experienced gene loss, a common phenomenon in mitochondrial genome studies (Adams et al. 2002; Adams and Palmer 2003; Li et al. 2023; Song et al. 2024). However, certain genes, including a core set of PCGs, 2 rRNAs, and even up to 38 tRNAs, were retained in the mitogenome, playing essential roles in cellular energy metabolism and environmental response (Allen 2015; Wang et al. 2020b). Similarly, to most fungi, the mitogenomes of the six *Diaporthe* species contained 15 PCGs (including *rps3* gene), 2 rRNAs, and 23 to 26 tRNAs, which appeared to be conserved when compared with other *Diaporthales* species (Suppl. material 1: table S2) (Kanzi et al. 2016; Koloniuk et al. 2016;) .Although the core PCGs in the six *Diaporthe* species were consistent in number and type, they displayed significant differences in sequence length, GC content, base composition, and codon usages. The impact of these variations on mitochondrial function requires further investigation. K2P results showed that the 15 core PCGs evolved at different rates, but all underwent purifying selection (Fig. 5). Non-conserved PCGs with unknown functions were also present in the mitogenomes of the six *Diaporthe* species, warranting further research to understand the origin and function of mitogenomes. Moreover, 14 out of the 23 tRNAs shared by the all *Diaporthe* species exhibited site variation (Suppl. material 1: tables S3, S4 and Fig. 2), suggesting that the frequency of mutations may influence fungal growth or stress responses. Mutations in tRNA were considered to be closely linked to protein synthesis efficiency and may ultimately impact the phenotype of eukaryotes (Giegé et al. 1998; Ding et al. 2019; Lin et al. 2021). The effects of tRNA mutations on *Diaporthe* species necessitate additional study. Additionally, the unique *trnI-2* (uau) presented in *D.sojae* VNIIKR SE Dps12, with an inserted fragment in the tRNA anticodon arm, but the effects of insertion fragment in tRNA on *D.sojae* VNIIKR SE Dps12 were unclear. Further investigation is required to understand the effects of the inserted fragment in tRNA on *D.sojae* VNIIKR SE Dps12.

### ﻿Dynamic changes of introns in *cox1* gene of Diaporthales

Introns were commonly found in fungal mitogenomes as mobile genetic elements, and their accumulation, movement and degeneration caused intron polymorphisms in different fungal species, affecting the organization and size of fungal mitogenomes (Hamari et al. 2002; Fan et al. 2019; Song et al. 2024). In the present study, we also observed that introns were the main factor contributing to the size variation of *Diaporthe* mitogenomes, as shown in Suppl. material 2: fig. S1. Further research had classified these introns into groups I and II, with group I introns being relatively abundant in fungal mitogenomes. Group I introns, containing homing endonucleases, were found to facilitate intron transfer (Megarioti and Kouvelis 2020). Previous studies have identified introns in various genes of fungal mitogenomes, including core PCGs and rRNA genes, with the *cox*1 gene being the primary host gene for fungal introns (Férandon et al. 2010; Ma et al. 2022). In our study, we also found that the *cox1* gene harbored the highest number of introns in the 13 *Diaporthales* mitogenomes, accounting for 31.11% of the total introns (Suppl. material 1: table S2). Further analysis of introns from the *cox1* gene revealed orthologous introns known as Pcls, which were useful for studying genetic variation within fungi and evolutionary relationships between species (Michel and Ferat 1995; Megarioti and Kouvelis 2020). In this research, we found that the number and type of Pcls in *Diaporthales* mitogenomes varied significantly, indicating frequent intron loss and gain events within *Diaporthales* species (Fig. 4 and Suppl. material 2: fig. S2). However, this variation appeared to be controlled within certain limits in mitogenomes of six species of the *Diaporthe* genus. P380, P877 and P1225 as universal Pcls were consistently detected in most *Diaporthe* species, while others (P554, P783, P899 and P975) were only once found in individual species (Fig. 4 and Suppl. material 2: fig. S2). This suggests that some universal introns may have been inherited from a common ancestor. In contrast, specific Pcls such as P554 and P975 were only detected in *D.eres* ZM79-3 and *D.sojae* VNIIKR SE Dps12, respectively, while homologous introns were detected in *Arthrobotrysoligospora* and *Erysiphenecator* far from the genus *Diaporthe*, indicating potential intron transfer events might have occurred between distant species. Future sequencing and analysis of additional mitogenomes from *Diaporthales* species are needed to uncover the mechanisms behind the origin, transfer, and evolution of these introns fully.

### ﻿Gene rearrangement of Diaporthales species

Mitochondrial gene arrangements offer valuable insights into genetic variation and phylogenetic relationships among different species (Satoh et al. 2010; Hikosaka et al. 2011; Zheng et al. 2018; Li et al. 2021). Currently, the study of mitochondrial gene rearrangements in fungi lags behind that in animals. While several models have been proposed to explain mitochondrial gene rearrangements in animals, a lot of studies have suggested that fungal mitogenomes may have more complex rearrangement mechanisms (Lavrov et al. 2002; Xia et al. 2016). Large-scale gene rearrangements were identified in 13 *Diaporthales* mitogenomes, yet six mitogenomes of *Diaporthe* species exhibited identical gene arrangements. Notably, similar gene arrangements were observed in different genera, such as *Cryphonectria* and *Chrysoporthe*, indicating a shared ancestry. Furthermore, a gene rearrangement involving the displacement of the *atp9* gene was found in four different genera, including *Diaporthe*, *Juglanconis*, *Cryphonectria*, and *Chrysoporthe* (Fig. 7). In contrast to the *Juglanconis* genus, *Ophiognomoniaclavigignenti-juglandacearum* displayed a distinct set of gene arrangements possibly inherited from unique ancestors. Previous studies suggested that the presence of repetitive DNA elements in intergenic regions played a significant role in mitochondrial gene rearrangements (Aguileta et al. 2014; Li et al. 2018a). However, despite the detection of numerous repeat elements in the six *Diaporthe* mitogenomes with content ranging from 1.41% to 10.99% (Suppl. material 1: table S7), no gene rearrangement was observed, consistent with findings in *Bipolaris* (Song et al. 2024). Therefore, factors beyond repetitive sequences may influence the gene rearrangement process in fungal mitogenomes. The mitogenomes of *Diaporthe*, *Juglanconis*, *Cryphonectria* and *Chrysoporthe­porthe* genera serve as typical examples offering valuable insights for further understanding the mechanism of mitochondrial gene rearrangement in fungi.

### ﻿Mitochondrial phylogenomic analysis

*Diaporthe* species are widely dispersed and can infect a variety of plant hosts, leading to significant economic losses (Bai et al. 2015; Zhao et al. 2022; Gao et al. 2024). Morphological variation within *Diaporthe* species has repeatedly been shown to be insufficient for species identification and defining novel species, as many closely related species or species complexes in the *Diaporthe* genus can be easily confused. Incorporating molecular data along with morphology is necessary to accurately describe species in the genus (Gao et al. 2017; Guo et al. 2020; Monkai et al. 2023). The introduction of molecular markers has advanced the taxonomy, population genetics, and biogeography of the genus *Diaporthe* (Gomes et al. 2013; Norphanphoun et al. 2022). But so far, it has been still often a hard work to distinguish the taxa of *Diaporthe* species accurately. In recent years, mitogenomes have been increasingly utilized in phylogeny and population study of plants, animals, and some kinds of fungi (Aguileta et al. 2014; Wang et al. 2020b; Song et al. 2024). In most cases, mitochondrial advantages over nuclear genomes, such as uniparental inheritance and accelerated evolutionary rates (Urantowka et al. 2017), have made mitogenomes a powerful tool in studying population genetics, taxonomy, and genetics in fungi (Wang et al. 2018; Johri et al. 2019; Ma et al. 2022). In this study, we constructed a highly supported phylogenetic tree containing 98 *Ascomycota* species based on the combined mitochondrial gene set and BI and ML analytical methods (Suppl. material 1: table S9 and Fig. 8). All these selected species were well gathered or divided into different independent clades and subclades, each of which exactly corresponded to one fungal taxa (such as class and order). The six *Diaporthe* species were observed to be closely related to species of the genera *Juglanconis*, *Ophiognomonia*, *Cryphonectria*, and *Chrysoporthe*, which was consistent with the previous analyses of multigene phylogeny based on five nuclear loci (Fan et al. 2018; Wu et al. 2019). Concluding, phylogenetic inference based on the combined mitochondrial gene set suggested that mitogenomes display a similar resolution power to classic phylogenetic analysis using nuclear DNA, potentially allowing promising applications for species delimitation within and between closely related genera of the genus *Diaporthe* and beyond in the future. However, the number of known mitogenomes in *Diaporthe* is still limited, considering the hundreds of described *Diaporthe* species. Further sequencing of the mitogenomes of closely related species in the genus *Diaporthe* is necessary to better understand the phylogenetic relationships and mechanisms of genetic variation within the genus.

## ﻿Conclusions

In the present study, the three newly sequenced *Diaporthe* mitogenomes (*Diaportheeres*, *D.phaseolorum* ZM33-4 and *Diaporthe* sp. ZM41-5) were assembled and compared with three previously sequenced *Diaporthe* mitogenomes (*D.caulivora* VNIIKR SE Dcaul3, *D.longicolla* MSPL 10-6 and *D.sojae* VNIIKR SE Dps12). Significant variation in size was observed among the mitogenomes of the six *Diaporthe* species, with the intronic regions contributing the most to mitogenome expansion. Comparative analysis revealed significant differences in gene contents, base compositions, gene lengths, tRNAs, and rRNAs among the six *Diaporthe* species. Large-scale gene rearrangements were found in the mitogenomes of 13 *Diaporthales* species with primary variations attributed to the position inversion of the *atp9* gene in different genera, while six mitogenomes of *Diaporthe* species had identical gene arrangements. The 15 core PCGs of the mitogenomes of 13 *Diaporthales* species exhibited different evolutionary rates but underwent conserved purifying selection throughout evolution. In addition, introns of the *cox1* gene in 13 *Diaporthales* mitogenomes experienced potential loss/gain, and transfer events, contributing to organization and size variations. Phylogenetic analysis demonstrated that mitochondrial genes could be used as a reliable tool to analyze phylogenetic relationships of *Diaporthe*. This study represents the initial exploration of the mitogenomes of *D.phaseolorum* ZM33-4 and *Diaporthe* sp. ZM41-5 It also marks the first comparative analysis of mitogenomes among species within the *Diaporthe* genus. These findings will enhance our comprehension of the genetics, evolution, and taxonomy of *Diaporthe* species.

## ﻿Additional information

### Adherence to national and international regulations

All the fungal strains used in this study have been legally obtained, respecting the Convention on Biological Diversity (Rio Convention).

### Author ORCIDs

Shunpei Xie https://orcid.org/0009-0009-9079-7829

Rui Zang https://orcid.org/0000-0001-8915-9250

Miao Liu https://orcid.org/0000-0003-3223-6010

Qingzhou Ma https://orcid.org/0000-0003-3716-9654

Yashuang Guo https://orcid.org/0000-0003-1299-6207

Meng Zhang https://orcid.org/0000-0003-1941-1136

### Data availability

All of the data that support the findings of this study are available in the main text or Supplementary Information.

## Supplementary materials

Supplementary material 1 Additional tables https://doi.org/10.3897/imafungus.16.140572.suppl1 Data type xlsx Explanation note **table S1**. Details of isolates of three *Diaporthe* used in this study. **table S2**. Comparison on mitogenomes among 13 species from *Diaporthales*. **table S3**. Annotation and characterization of the six *Diaporthe* mitogenomes. **table S4.** Distribution details of mutation sites in tRNA genes of six *Diaporthe* mitogenomes. **table S5**. Core protein coding gene information of the six *Diaporthe* species. **table S6**. Codon usage analysis of the six *Diaporthe* mitogenomes. **table S7**. Local BLAST analysis of the six *Diaporthe* species mitogenomes against themselves. **table S8**. Tandem repeats detected in the mitogenomes of the six *Diaporthe* species using the Tandem Repeats Finder. **table S9**. Species, and GenBank accession number used for phylogenetic analysis in this study. https://binary.pensoft.net/file/1265892This dataset is made available under the Open Database License (http://opendatacommons.org/licenses/odbl/1.0/). The Open Database License (ODbL) is a license agreement intended to allow users to freely share, modify, and use this Dataset while maintaining this same freedom for others, provided that the original source and author(s) are credited.Shunpei Xie, Xuyang Ma, Haiyan Wu, Rui Zang, Haiqiang Li, Miao Liu, Qiang Li, Qingzhou Ma, Yashuang Guo, Meng Zhang

Supplementary material 2 Additional figures https://doi.org/10.3897/imafungus.16.140572.suppl2 Data type pdf Explanation note **fig. S1** The proportion of different genetic compositions and their contribution to mitogenome expansion (above) and contraction (below) in six *Diaporthe* mitogenomes. **fig. S2** Insertion sites of different position classes (Pcls) in the coding regions of *cox1* genes of 13 species. Protein sequences encoded by the *cox1* genes of 12 other species were aligned with the *cox1* of *Juglanconisjuglandina*. The Pcls were named according to their insertion sites in the reference *cox1* sequence of *J.juglandina*. The symbols ‘+1’ and ‘+2’ refer to the different insertion positions of Pcls within triplet codons: ‘+1’ when between the 1^st^ and 2^nd^ nt of a codon; and ‘+2’ when between the 2nt and 3nd nt of a codon. **fig. S3** Collinearity analysis of six *Diaporthe* mitogenomes as generated with Mauve 2.4.0. Homologous regions between different species were represented by the same color blocks and connected by the same color lines. https://binary.pensoft.net/file/1265893This dataset is made available under the Open Database License (http://opendatacommons.org/licenses/odbl/1.0/). The Open Database License (ODbL) is a license agreement intended to allow users to freely share, modify, and use this Dataset while maintaining this same freedom for others, provided that the original source and author(s) are credited.Shunpei Xie, Xuyang Ma, Haiyan Wu, Rui Zang, Haiqiang Li, Miao Liu, Qiang Li, Qingzhou Ma, Yashuang Guo, Meng Zhang
