# Supplementary material for: ﻿The six whole mitochondrial genomes for the Diaporthe species: features, evolution and phylogeny
Source: IMA Fungus. 2025 Feb 28;16:e140572. doi: 10.3897/imafungus.16.140572 (PMC11889515; doi:10.3897/imafungus.16.140572)
Supplement: Supplementary material 2 — Additional figures [file imafungus-16-e140572-s002.pdf]

Suppl. material 2

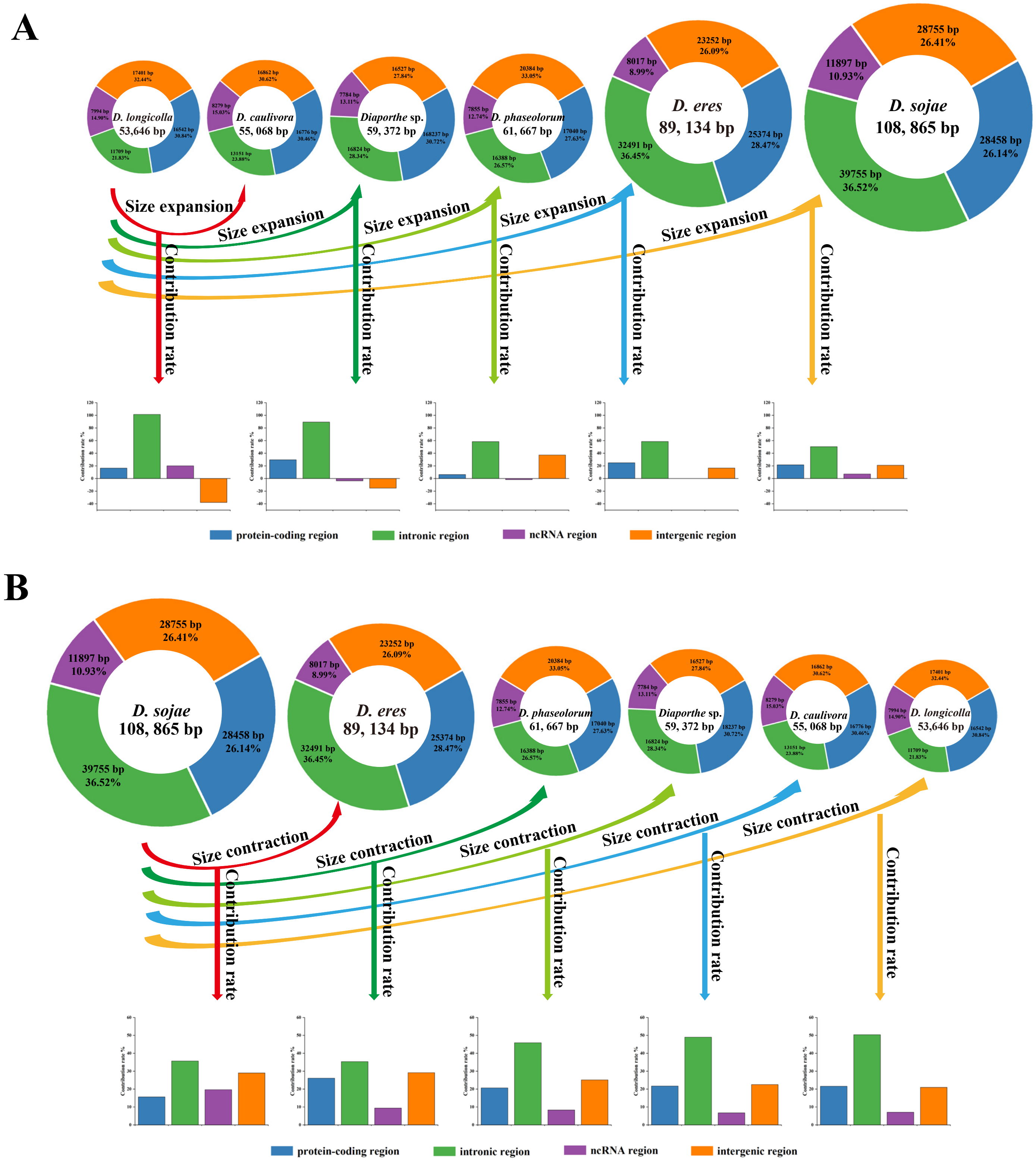

figure S1. The proportion of different genetic compositions and their contribution to mitogenome contraction (above) and expansion (below) in six *Diaporthe* mitogenomes.

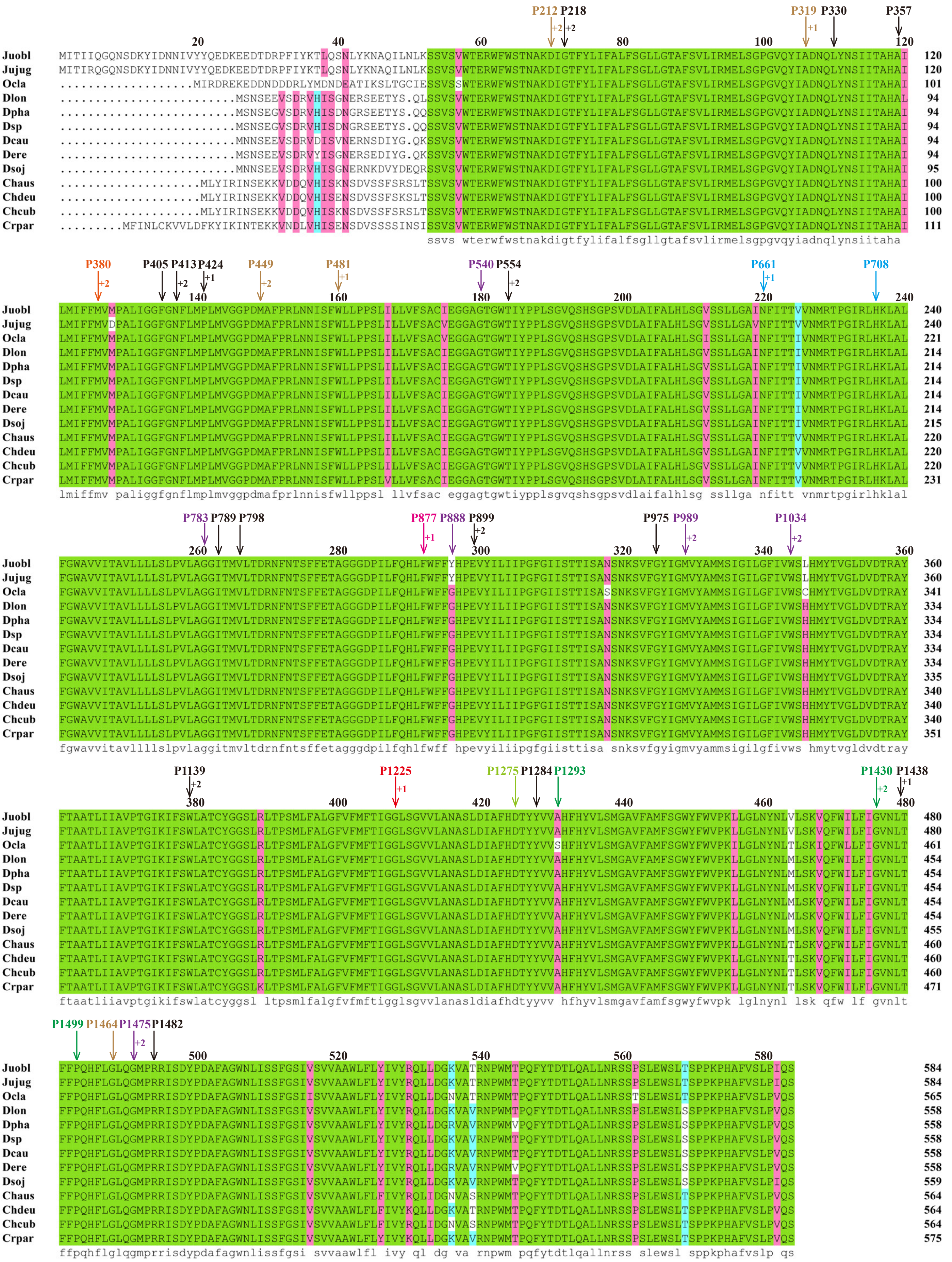

figure S2. Insertion sites of different position classes (Pcls) in the coding regions of *cox1* genes of 13 species. Protein sequences encoded by the *cox1* genes of 12 other species were aligned with the *cox1* of *J. juglandina*. The Pcls were named according to their insertion sites in the reference *cox1* sequence of *J. juglandina*. The symbols '+1' and '+2' refer to the different insertion positions of Pcls within triplet codons: '+1' when between the 1st and 2nd nt of a codon; and '+2' when between the 2nt and 3nd nt of a codon.

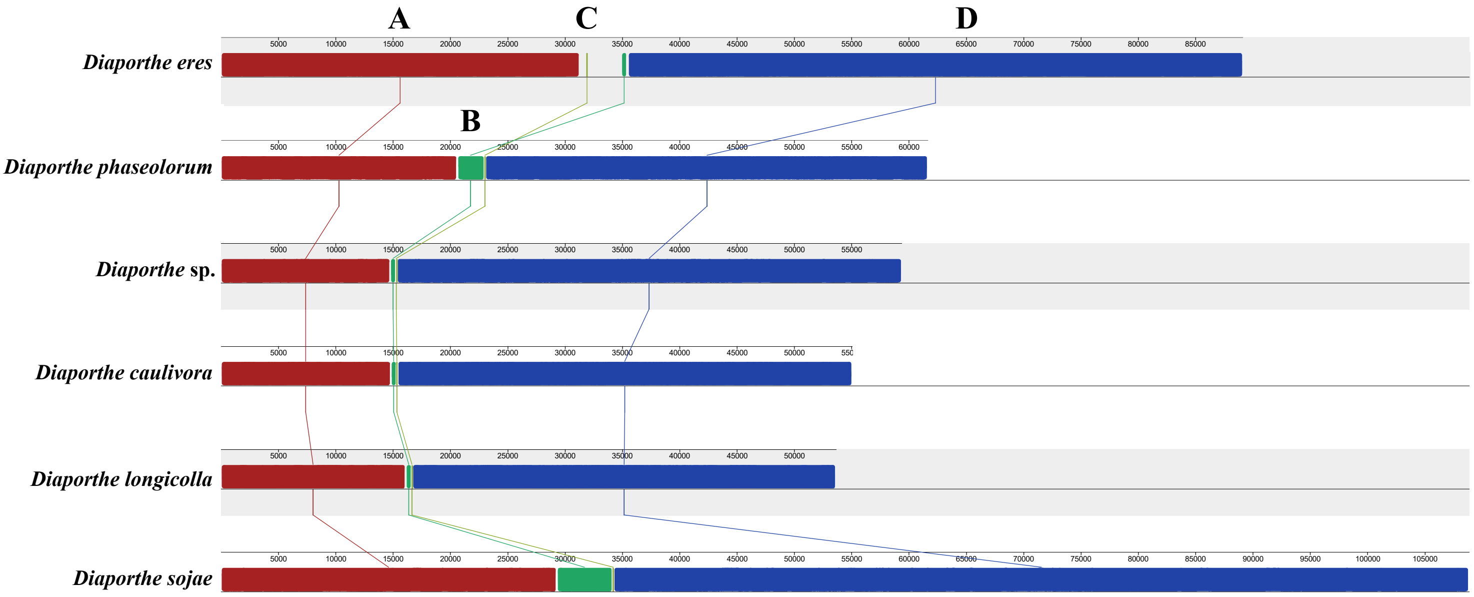

figure S3. Collinearity analysis of six *Diaporthe* mitogenomes as generated with Mauve 2.4.0. Homologous regions between different species were represented by the same color blocks and connected by the same color lines.
